# Acute appendicitis, inflammatory appendiceal mass and the risk of a hidden malignant tumor: a systematic review of the literature

**DOI:** 10.1186/s13017-017-0122-9

**Published:** 2017-03-09

**Authors:** Frederico José Ribeiro Teixeira, Sérgio Dias do Couto Netto, Eduardo Hiroshi Akaishi, Edivaldo Massazo Utiyama, Carlos Augusto Metidieri Menegozzo, Marcelo Cristiano Rocha

**Affiliations:** 1 Surgical Oncology Group from the III Surgical Clinic Division, Hospital das Clínicas of the University of São Paulo (USP), School of Medicine, Rua Dr. Ovídio Pires de Campos, 255, 8 th floor, room 8131, Cerqueira César, 05403-010 São Paulo Brazil; 2 Surgical Oncology Group from the III Surgical Clinic Division, Hospital das Clínicas of the University of São Paulo (USP), School of Medicine, Rua Dr. Ovídio Pires de Campos, 255, 8 th floor, room 8131, Cerqueira César, 05403-010 São Paulo Brazil; 3 Emergency Surgical Service, Hospital das Clínicas of the University of São Paulo (USP), School of Medicine, Rua Dr. Ovídio Pires de Campos, 255, 8 th floor, room 8131, Cerqueira César, 05403-010 São Paulo Brazil; 4 Sarcoma and Melanoma Surgery group - São Paulo Cancer Institute, University of São Paulo, School of Medicine, Cerqueira César, Brazil; 5 General and Trauma Surgery - III Surgical Clinic Division, Hospital das Clínicas of the University of São Paulo (USP), School of Medicine, Rua Dr. Ovídio Pires de Campos, 255, 8 th floor, room 8131, Cerqueira César, 05403-010 São Paulo Brazil; 6 850, Francisco Matarazzo Avenue, apt 181, Bloco 2, Zip Code 05001-200 Perdizes, São Paulo Brazil; 7 General Surgery Senior Resident - III Surgical Clinic Division, Hospital das Clínicas of the University of São Paulo, School of Medicine, São Paulo, Brazil; 8 Chief of Staff of the Emergency Surgical Service - III Surgical Clinic Division, Hospital das Clínicas of the University of São Paulo, School of Medicine, São Paulo, Brazil

**Keywords:** Appendiceal inflammatory mass, Acute appendicits, Appendiceal neoplasms, Appendiceal neuroendocrine tumors, Pseudomixoma peritonei, Interval appendectomy

## Abstract

**Introduction:**

Acute appendicitis is significantly common. Despite the increased use of computed tomography, the number of perforated cases has been stable in the past three decades. Between 2% and 6% of patients with acute appendicitis present appendiceal mass, often described as inflammatory phlegmon or abscess. Malignant tumors are confirmed by pathological analysis in 0.9–1.4% of all appendectomies performed to treat acute appendicitis. However, recent series demonstrate an elevated incidence of malignancies, ranging from 5.9 to 12%, in patients with inflammatory appendiceal mass.

**Methods:**

The analysis was based on a systematic review of the literature. The articles were searched in PubMed for the period from 1987 to 2016. Articles presenting the incidence of the hidden malignancy among patients with appendiceal inflammatory mass were selected. Variables as age, interval appendectomy rate, the incidence of neoplasm, time to surgery, minimally invasive assessment, histology, right colectomy rate and morbidity were analyzed.

**Results:**

A total of 13.244 patients were described as presenting acute appendicitis. Appendiceal tumor is present in approximately 1% of the appendectomies, while the rate of neoplasm varies from 10 to 29% in patients presenting appendiceal inflammatory mass. Interval appendectomies, despite been the minority of the procedures, disregard the higher morbidity associated with right sided colectomies. The review of literature also describes oncologic, histologic and clinical aspects of patients presenting appendiceal neoplasm, describing the most frequent histologic subtypes of this illness.

**Conclusion:**

Hidden appendiceal neoplasm in acute appendicitis are rare, fortunately. However, its incidence is much higher in patients presenting appendiceal inflammatory mass. Hence, interval appendectomy should be considered in this subgroup of patients.

## Background

The incidence of appendicitis demanding emergency surgical treatment is significantly common, with appendectomy being used as the standard treatment since 1889 [1]. Only in the US, appendicitis generates about 300,000 annual hospital admissions [2].

Despite the increased use of computed tomography to evaluate acute abdominal pain and diagnose appendicitis, and of laparoscopic appendectomy, the number of perforated cases has been stable in the past three decades. Between 2 and 6% of patients with acute appendicitis present appendiceal mass, often described as inflammatory phlegmon or abscess [3]. In these cases, the ideal approach is still controversial, since there are proposals to an indirect, non-surgical treatment.

When conservative treatments have a positive result at first, there is often a dilemma whether or not to perform an interval appendectomy or maintain nonoperative approach. However, the real disadvantage of the latter is not having the appendix submitted to histological analysis. In a meta-analysis and systematic review of 2771 patients diagnosed with inflammatory appendiceal mass (phlegmon or abscess), Andersson and collaborators found 31 with malignant tumors [4].

These lesions are detected in 0.9 to 1.4% of the appendectomies performed to treat acute appendicitis [5, 6].

Although rare, appendiceal tumors represent about 1% of malignancies of the large intestines. Acute appendicitis is the initial presentation of primary neoplasms of the appendix in more than 50% of the cases, causing an unpleasant surprise to both the doctor and the patient [7].

It’s reported that 2 to 6% of acute appendicitis cases consist in inflammatory appendiceal mass [8], and recent series demonstrate an elevated incidence of malignant tumors in this organ, between 5.9 and 12% [9, 10].

## Methods

The present analysis was made through a Medline search with the profiles “acute appendicitis AND (conservative OR interval appendectomy OR mass OR abscess OR phlegmon)”, and identified references, written in English, between 1987 and 2016.

Inclusion criteria for review consisted in searching articles describing patients older than 18 years-old treated for acute appendicitis with abdominal abscess or phlegmon who underwent, or not, interval appendectomy, with pathological analysis of the appendix described.

Based on the title and the abstracts, potential articles were selected, and the full text was analyzed to identify original studies that reported results from this treatment. Variables as age, interval appendectomy rate, the incidence of neoplasm, time to surgery, minimally invasive assessment, histology, right colectomy rate and morbidity were analyzed.

A review of the literature regarding the main clinical presentation, oncologic, histopathologic features was made. In this context, surgical management of appendiceal neoplasms was also highlighted.

## Results

**Fig. 1:**
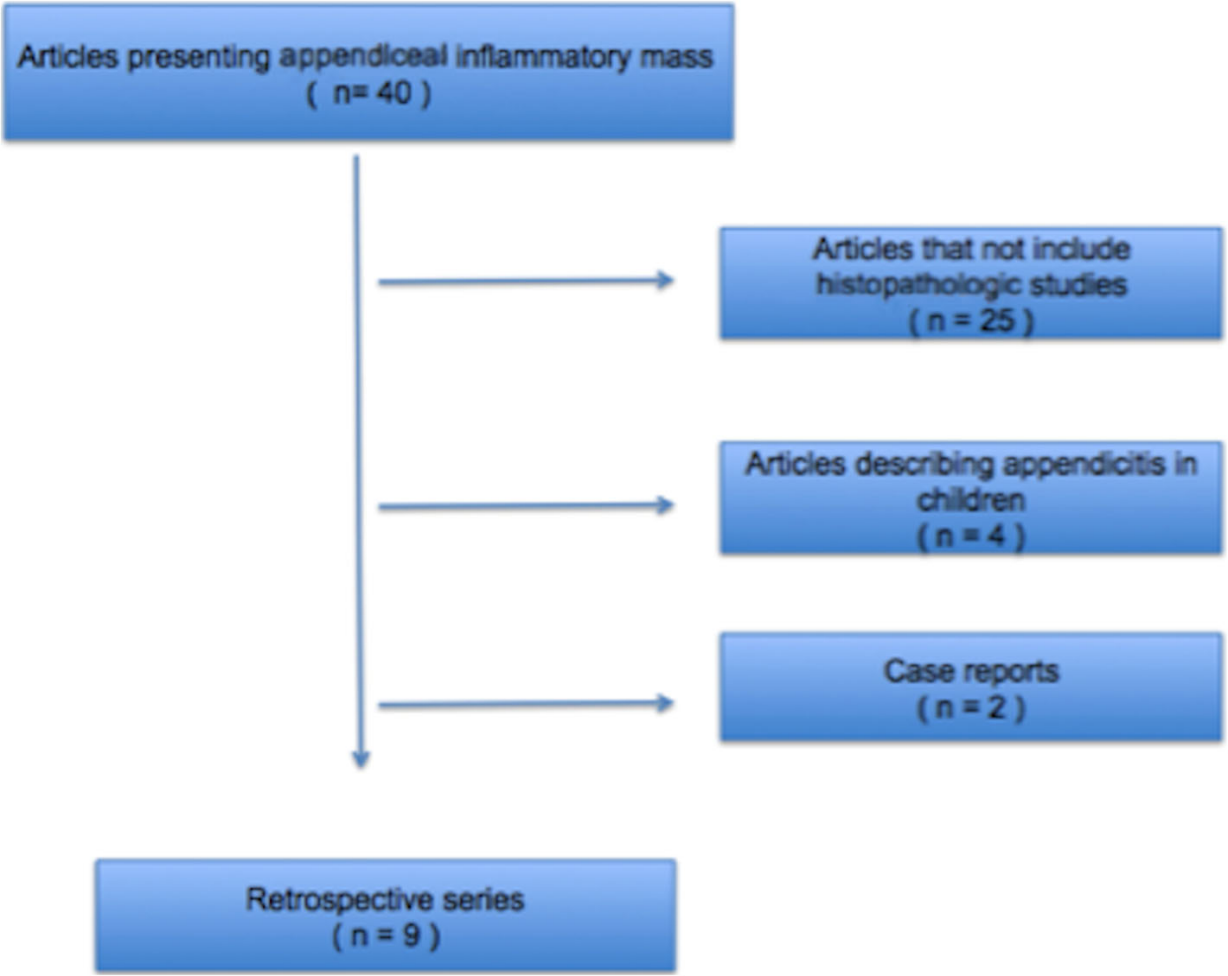
Diagram of study selection

A total of 40 initial reports were identified. Four were excluded because they described interval appendectomy in children. Seventeen studies were excluded because, despite being systematic reviews or retrospective case series, they did not contemplate histologic features from operated patients. Two other papers were excluded because they were case reports of only one patient. A total of nine uncontrolled retrospective series fulfilled the inclusion criteria (Fig. 1). A total of 13.244 patients were described as presenting acute appendicitis.

Eight studies reported the mean ages of patients showing appendicitis [8, 11–17]. Patients presenting with appendiceal abscess who underwent interval appendectomy were older than those submitted to an upfront appendectomy. The mean age of patients with usual appendicitis was 49.7 ± 6 years old (ranging from 41 to 57 years-old). Of those with inflammatory mass, average age was 52.4 ± 5.9 years old (ranging from 46 to 62 years-old) and 52% of them were male. The majority of patients was initially managed with immediate appendectomy or conservative treatment (77,5 and 16% respectively). Only a few patients were subjected to an interval appendectomy, in a mean of 6.5% in pooled analysis [8, 11–16, 18]. Six papers reported interval appendectomy rates that varied from 1.5% to 28% [8, 16], and five of them addressed less than 10% of patients been treated by interval procedure [8, 11, 13, 14]. Even Tingstedt B. et al [16] states the changes in treatment choices throughout the decades. Interval appendectomy was very common before 1995, in up to 46% of the patients, showing a reduction to 8% after that period.

Laparoscopic appendectomy was less frequent for interval appendectomy; only two papers [8, 11] were describing surgical technique. Minimally invasive approach for appendiceal mass ranged from 54 to 78% compared to 99%, from the same author, when treating noncomplicated appendicitis. The rates of conversion to open procedures were also higher in appendiceal mass than usual, varying from 8 to 29% [11, 18].

Four articles [8, 11, 13, 14] described the time to surgery, which ranged from 52 to 78 days, with a mean of 63 ± 11 days.

No patient had appendiceal neoplasm diagnosed preoperatively, and overall incidence was extremely low. From 13.244 patients operated from appendicitis, 206 were submitted to interval appendectomy, 24 underwent right colectomy, and 130 (0.9%) had the histopathological analysis demonstrating tumor [8, 11–16, 18]. The incidence ranged from 0.3 to 3.2%. However, in patients presenting with appendiceal inflammatory mass the rate of neoplasm was much higher, from 10 to 29%, as stated in Table 1. The incidence of tumor’s histological subtypes is described in Table 2.

**Table 1:** Articles considering incidental neoplasm findings among appendiceal abscess

Author	Year	Appendicitis (N)	Abscess	Neoplasm N (%)
Carpenter S.G. et al.	2012	315	18	5 28
Lee W.S. et al.	2011	3744	(−)	28 (−)
Roberts J.K. et al.	2008	876	41	4 10
Wright G.P. et al.	2015	6038	82	11 12
Deelder J.D. et al.	2014	(−)	119	12 10
Lai H.W. et al.	2006	1873	70	7 10
Furman M.J. et al.	2013	376	17	5 29
Cerame M.A. et al.	1987	305	192	56 29
Tingstedt B. et al.	2002	93	30	3 10
Sum		13.244	569	131 23%

**Table 2:** Incidence among histologic subtypes

Author	Histologic subtypes	% (N)
Carpenter S.G. et al. [11]	LGMN	0%
	Mucinous AdenoCa	20% (1)
	In situ AdenoCa	20%(1)
	Adenocarcinoma	40%(2)
	Carcinoid	20%(1)
Lee W.S. et al. [12]	LGMN	50%(14)
	Mucinous AdenoCa	11%(3)
	In situ AdenoCa	0%
	Adenocarcinoma	7%(2)
	Carcinoid	32%(9)
Wright G.P. et al. [8]	LGMN	
	Mucinous AdenoCa	55%(6)
	In situ AdenoCa	0%
	Adenocarcinoma	9%(1)
	Carcinoid	36%(4)
Lai H.W. et al. [13]	LGMN	80%(5)
	Mucinous AdenoCa	0%
	In situ AdenoCa	0%
	Adenocarcinoma	20%(2)
	Carcinoid	
Furman M.J. et al. [14]	LGMN	100%(5)
	Mucinous AdenoCa	
	In situ AdenoCa	
	Adenocarcinoma	
	Carcinoid	
Cerame M.A. et al. [15]	LGMN	0%
	Mucinous AdenoCa	40%(79)
	In situ AdenoCa	1%(2)
	Adenocarcinoma	59%(117)
	Carcinoid	
Tingstedt B. et al. [16]	LGMN	33%(1)
	Mucinous AdenoCa	0%
	In situ AdenoCa	0%
	Adenocarcinoma	66%(2)
	Carcinoid	0%

Interval appendectomy shows morbidity varying from 6 to 11% [8, 13, 14]. The most frequent complications were assigned as minor representing wound infections, but there were post-operative abscesses, colocutaneous fistulas and hemorrhage [8]. Right-side hemicolectomy was employed in 7 to 25% of the cases [8, 12], and there was one fatal complication due to anastomotic leakage [8].

## Review of the literature

### Classifying and staging cecal appendix tumors

Recognizing the differences in the clinical presentation and the prognosis of appendiceal primary tumors is essential. When the tumor presents a signet-ring cell formation, the average survival is 6 months. Meanwhile, when histology shows a low-grade mucinous neoplasm, the median overall survival increases to 8 years [17].

**Fig. 2:**
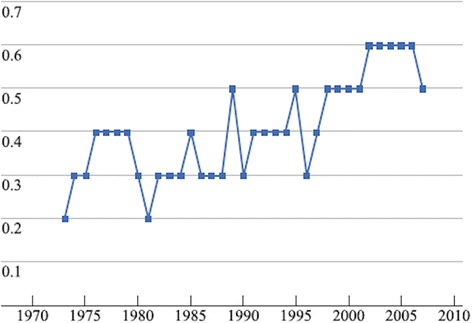
The number of appendix tumors, adjusted by age, from 1997 to 2007, according to SEER database (per 100,000 inhabitants)

Recently, a study based in the database produced by the Surveillance, Epidemiology and End Results (SEER) of the National Institute of Cancer of Bethesda revealed different survival rates for 5655 patients diagnosed with a primary appendiceal tumor, depending on the histology. The same study also showed an increased number of accidental diagnosis, which can be explained by a boost in the number of patients submitted to computed tomography and by the substantial use of laparoscopy, improving the sight of the abdominal cavity [19] (Fig. 2).

**Fig. 3:**
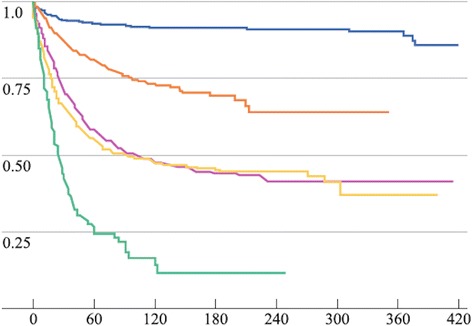
Survival according to the histological type. Modified by Turaga et cols, Ann SurgOncol (2012) 19:1379–1385

The overall survival rate related to the disease in the five coming years changed in line with the histologic subtype [19] (Fig. 3; Table 3).

**Table 3:** Specific survival rate of the disease in the coming five years according to its histologic subtype

Histologic subtype.	Survival rate per specific disease in 5 years %.
Carcinoid tumor	91%
Globet cell carcinoid	81%.
Mucinous adenocarcinoma	58%
Non-specificadenocarcinoma	55%
Signet-ring cell formation.	27%

The World Health Organization (WHO) classifies appendiceal tumors in two main groups: appendiceal carcinomas and neuroendocrine neoplasia (Table 4).

**Table 4:** The World Health Organization classification (2010)

Appendiceal carcinomas.	
• Adenocarcinoma 8140/3	
• Mucinous adenocarcinoma 8480/3	
• Low-grade mucinous neoplasia 8480/1	
• Signet-ring cell formation carcinoma 8490/3	
• Undifferentiated carcinoma 8020/3	
Neuroendocrine appendix neoplasias.	
• Neuroendocrine tumors (NET).	
• NET G1 (carcinoid) 8240/3	
• NET G2 8249/3	
• Neuroendocrine carcinomas (NEC) 8246/3	
• NEC large cells type 8013/3	
• NEC smal cells type 8041/3	
• Mixed tumor:	
• Mixed Adenoneuroendocrine carcinomas 8244/3	
• Serotonin-producing endocrine carcinoma 8241/3	
• Globet cell carcinoid 8243/3	
• Tubular carcinoid 8245/1	
• Peptide-producing/ Glucagon-producing large cell tumor PP/PYY 8152/1	

### Appendiceal neuroendocrine (NET) tumors

Oberndorfer first described NET tumors in the ileum and appendix in 1907, with mention to an indolent course, although some of them, with a different phenotype, had a more unfavorable outcome [20]. The two sub-groups of carcinoids based on their histological characteristics are Enterochromaffin cell serotonin producing carcinoid and its tubular variation with L-cell glucagon-like peptide, and PP/PPY producing tumor.

Therefore, carcinoid is a definition mostly used for tumors with more favorable histological characteristics while neuroendocrine is a broader description, which includes more aggressive attributes of the disease.

### Epidemiology and prognosis

NETs have approximately 0.15 per 100,000/year incidence, according to the SEER registry. The frequency is similar to other populations, based on European databases [21–24].

Those NETs are frequent when considering only neuroendocrine gastroenteropancreatic tumors. There is slight prevalence among women, affecting mostly Caucasians, and its incidence could be underestimated, since most of the cases, which are incidentally diagnosed, may not be notified in tertiary centers. The frequency of appendectomies performed without any particular reason is 3–9 per 1000 inhabitants [25, 26].

Appendiceal NET belongs to a sub-group of neoplasia with a significant number of incidental diagnosis, representing about 80% of the neoplasia, including benign and malignant tumors. The average age of the diagnosis varies from 38 to 51 years old [27–29].

In most cases, the prognosis is excellent, with 100% of overall 5-year survival rate for localized disease and 85–100% for regional disease. Nevertheless, when including staging, the 5-year survival rates vary between 70 and 85%. Advanced disease has unfavorable prognosis with a 5-year survival rate of 12 and 28%. Tumors with more aggressive histology, such as Goblet cell carcinoids and mixed adenoneuroendocrine carcinomas, were not included in this study [27].

**Fig. 4:**
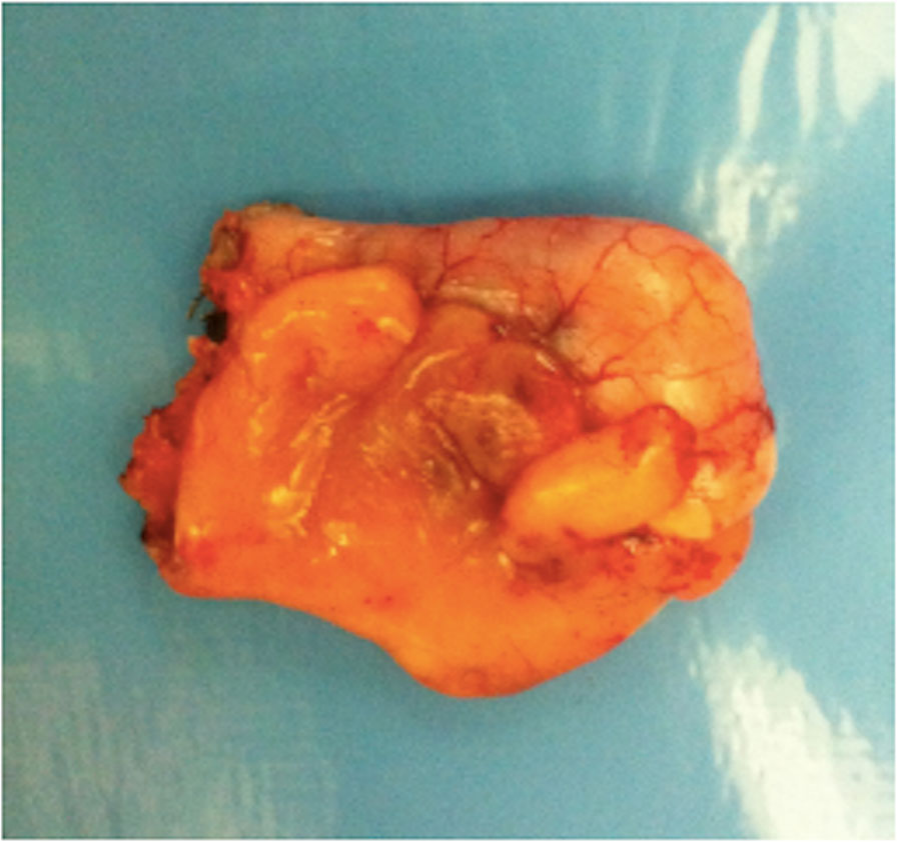
Well-differentiated neuroendocrine neoplasia, with 1 to 2 cm, located in the tip of the appendix. Degree I (WHO) (Courtesy of Andre Bouzas, MD – Oncology Institute - Santa Paula Hospital, São Paulo

For NETs, Location, tumor depth, dimensions and possible invasion of the mesoappendix are important factors to calculate disease recurrence. In 70% of the cases, the tumors are located at the tip of the appendix, leading to a lower recurrence rate (Fig. 4). About its length, tumors with 1 cm (60 to 80% of the cases), invading through the subserosa or into the mesoappendix for 3 mm also have a lower recurrence rate. However, tumors with positive margins, located in the appendicular base, larger than 2 cm, or deeply invading the mesoappendix, have a higher recurrence rate, suggesting the need to perform additional resection [30–32].

The presentation of the disease is usually associated with acute appendicitis. It rarely evolves into a metastatic disease and hardly ever develops carcinoid syndrome.

Because there is no suspicion of NET before an appendectomy is performed, preoperative exams do not offer additional information. Computed tomography or nuclear magnetic imaging could be used in postoperative exams, to clinically stage the regional (lymph nodes) or metastatic disease. Usually, colonoscopy will not reveal the tumor, since in 70% of the time it occurs in the tip of the appendix, rarely invading the caecum. Metabolic studies, such as Octreo Scan and gallium 68 PET-CT, are unusually performed. Laboratory tests, such as serum chromogranin A and 5-hydroxyndoleaticeticacid, are used selectively in the follow-up, or for suspicion of possible carcinoid syndrome, respectively. A well-differentiated NET, smaller than one centimeter and treated with R0 resection, usually do not evolve into a metastatic or regional disease in follow-up. Postoperative computed tomography and MRI are justified when tumors measure 1 to 2 cm. In cases of larger tumors, deep invasion of the mesoappendix or angiolymphatic invasion, image-led exams and metabolic tests, such as OctreoScan and gallium 68 PET-CT, are often required.

The histopathological test should be revised and must describe location of the tumor, grade, mitotic index, Ki-67 rate, dimension of the tumor, potential multifocal/ multicentric disease, vascular invasion, perineural invasion, presence of non-endocrine component, presence and number of lymph nodes, margin status, and TNM system (Table 5).

**Table 5:** TNM staging

Neuroendocrine tumors:	
T:	
Tx: Primary tumor which cannot be evaluated.	
T0: No primary tumor was found in the appendix.	
T1: Tumor with up to 2 cm dimension.	
T1a: Up to one centimeter.	
T1b: Up to 2 cm.	
T2: Greater than two up to 4 cm or with extensions to the caecum.	
T3: Greater than 4 cm or extension to the ileum.	
T4: Tumor invades abdominal wall or adjacent organs or perforated tumor	
N:	
NX: Nodal staging could not be evaluated for lack of information.	
N0: Absence of metastasis in regional lymph nodes	
N1: Presence of metasis in regional lymph nodes	
M:	
M0: Absence of distant metastasis	
M1: Presence of distant metastasis	
Stadium I: T1N0M0.	
Stadium II: T2/T3N0M0.	
Statium III: TN1M0 / T4N0M0.	
Statium IV: TNM1.	

Individual immunohistochemical studies could be included to detect neuroendocrine markers, including chromogranin A, synaptophysin, specific peptides and others.

Tumor grade is determined by the mitotic index and Ki-67 (Table 6).

**Table 6:** Grade of NET tumors according to the World Health Organization (WHO), considering the Mitotic index and Ki-67

Grade	NETs appendix	Tumor differentiation
Low grade (G1)	<2 mitoses /10 high power fields (HPF) or < Ki-67 < 3%.	Well-differentiated
Intermediate grade (G2)	2–10 mitoses / 10 HPF or Ki-67 between 3 and 20%.	Well-differentiated
High grade (G3)	>10 mitoses / 10 HPF or Ki-67 > 20%.	Poorly differentiated

A major factor in the grading risk is the accurate staging of T in the TNM system since the diagnosis is usually incidental. For tumors T1a (UICC/AJCC), a simple appendectomy is appropriate treatment, also in the pediatric population. The overall survival in this group is 100% [33, 34].

Tumors larger than 2 cm (T2) are rare (<10%), but carry a risk of becoming a metastatic disease in 25 to 40% of the cases. Because of such previous data, radical resection is indicated, with follow-up for high-risk disease [35–39].

Other criteria also considered is the location of the tumor in the cecal appendix. Most of the tumors (60 to 75%) are located in the tip of the appendix, 5 to 20% in the middle third and less than 10% in the appendiceal base. It has been suggested metastasis frequently appear in tumors located at the base, suggesting additional resection when the tumors have 1 to 2 cm length.

The decision to perform additional resection depends on the extent of mesoappendix invasion, if present. Up to 20% of the adults and 40% of the children undergo further resection. It is possible that the invasion of the mesoappendix be greater than initially reported. Despite not being considered in the TNM staging, the invasion of the mesoappendix could be related to a higher appearance of lymphatic invasion. When the malignancy compromises more than 3 mm, there is increased risk of nodal metastasis, and additional resection should be considered [40, 41].

### Surgical treatment

Resection options for NET are appendectomy, ileocolectomy, and right colectomy. For T1a tumors with no invasion of the mesoappendix or not located in the appendiceal base, an appendectomy should suffice. When the tumor has invaded the mesoappendix in more than 3 mm, or the tumor is located in the base, an ileocolectomy and right colectomy should be indicated. However, there is not enough data to support the hypothesis that an additional resection will increase patient survival and its morbidity should also be considered.

When tumors have dimensions greater than 2 cm, the ileocolectomy or right colectomy should be performed. For those between 1 and 2 cm, other criteria are considered before performing additional resection, including age, extension of mesoappendix invasion, and location. However, there is scarce long-term follow-up information of the patients. Angioinvasion or G2 could also be taken into consideration when deciding for additional resection. However, there is even less evidence published about such criteria. Nevertheless, additional resection has been used based on the consensus of the European Neuroendocrine Tumor Society and when certain histologic characteristics are present.

### Carcinoid/Globet cell carcinoid

It is a mixed phenotype tumor, with goblet cell morphology and neuroendocrine features more or less pronounced. The incidence of such tumor is extremely rare, representing less than 5% of primary appendiceal tumors. The disease is common among Caucasians, who represent 80% of the studied population [42].

The overall 5-year survival rate is lower than well-differentiated NET and varies between 40% and 75%. The disease can behave as adenocarcinomas with more or less pronounced phenotype of goblet cells or neuroendocrine tumors [43]. In two-thirds of the goblet cell tumor patients, the diagnosis is incidental, resulting from an appendectomy for acute appendicitis, which represents 50 to 60% of the initial presentation. Ten to 20% of them present peritonitis resulting from perforation [44, 45].

In 50% of the cases, the tumor invades the serosa and the mesoappendix. In 15 to 30% of the patients, there is the occurrence of distant metastasis, commonly in the ovaries, or in the lymph nodes. However, when measurement is possible, the progression of the disease is increased when tumors have more than 2 cm in length.

Other parameters foreseeing the aggressive behavior of the illness include high mitotic counts (more than two mitoses per 10 high-power fields); a Ki-67 greater than 3%; invasion into the serosa and into the mesoappendix; angioinvasion; increased number of Paneth cells; enhanced production of mucin; production of pancreatic polypeptide and lymph node involvement [46, 47].

In a retrospective series of 63 patients with carcinoid/goblet cell carcinoid, Tang and collaborators demonstrated the correlation between the pathological analysis and the, aggressiveness of the disease. According to the morphologic characteristics, three subgroups were found [48]:

- Group A –Typical Goblet cell carcinoid. Tumors had minimum cellular atypia, rare mitoses and minimum stromal invasion. The overall survival rate in 5 years was 100%.
- Group B – Goblet cell adenocarcinoid and signet-ring cell formation. Tumors presenting an evident cellular atypia, prominent desmoplasia and structural distortion of the appendiceal wall. Overall survival rate in 5 years was 36%.
- Group C – Undifferentiated Adenocarcinoma with goblet cell carcinoid. The overall survival rate in 5 years was 0%.

### Epithelial tumors of the appendix

Merling published the first reference to appendiceal neoplasms in 1838. In 1903, Elting published a series of patients with tumors emerging from preexisting adenomas in the appendix, assuming a growth pattern with cystic dilatation of the appendix (mucinous tumors). He realized that sometimes it could exhibit a growth pattern similar to primary colon tumors (colonic-types), these are either polypoid or ulcerative, emerging from tubular or tubulovillous adenomas [49].

The mucinous-type is inclined to rupture and mucin extravasation, with or without mucin epithelia into the abdominal cavity, resulting into Pseudo myxoma Peritonei [42, 50]. The Colonic-type is similar to colon adenocarcinomas and results in lower overall survival rate than mucinous-type tumors [51].

In an analysis based on the SEER registry between 1973 and 2004, 2791 patients with malignant appendiceal neoplasm were studied. Adenocarcinomas were more frequent, responding for 65.4% of the cases. While neuroendocrine tumors have presented a regular occurrence in the same period, there was a 260% increase in carcinoma rates. The overall 5-year survival rate for those patients was 46.5%. When considering mucinous cystadenocarcinoma, it rose to 59% in all stages, and in signet-ring cell formation tumors, to 20.3% [52].

### Mucinous appendiceal tumors

Although appendiceal mucinous tumors are recognized as benign, as per their histology, those tumors can progress to peritoneal dissemination.

Ronnet and collaborators, in 1995, published a classification based on 109 patients who presented peritoneal dissemination caused by mucinous epithelial tumor in appendix [53].

- Disseminated Peritoneal Adenomucinosis (DPAM): Peritoneal lesions composed of abundant extracellular mucin, with minimum cytologic atypia and rare mitoses. In all the cases, the appendix tumor was an adenoma
- Peritoneal Mucinous Carcinomatosis (PMCA): Peritoneal lesions composed of more abundant mucinous epithelium with the architectural and cytologic features of carcinoma. The primary tumor was mucinous appendix carcinoma, with prominent cytologic atypia and, in some cases, with signet-ring cell formation. The distinct subgroup can potentially evolve to metastatic disease to other organs or lymph nodes.

**Fig. 5:**
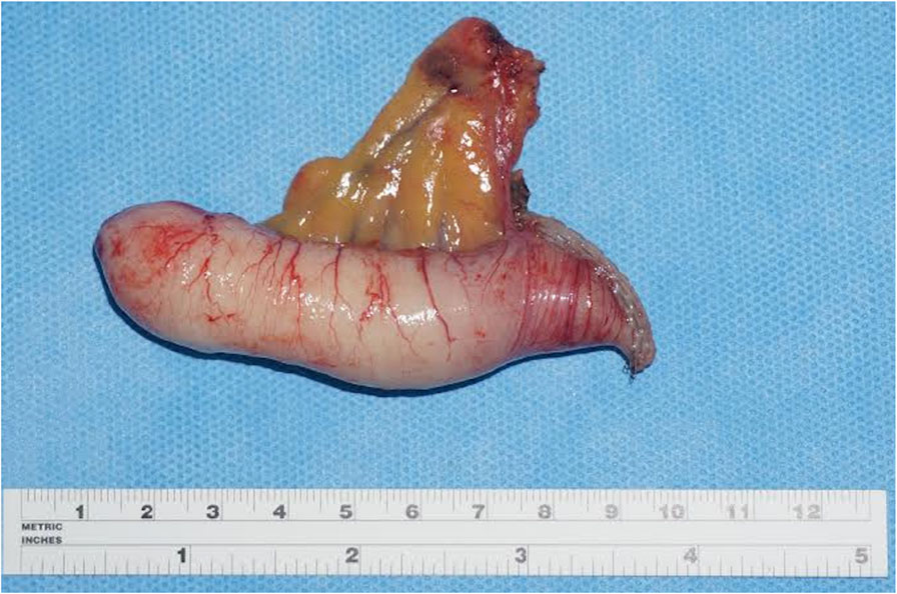
Low-grade mucinous neoplasia (LAMN – Grade 1 WHO), measuring 2 cm, invading the distal third with free margin

**Fig. 6:**
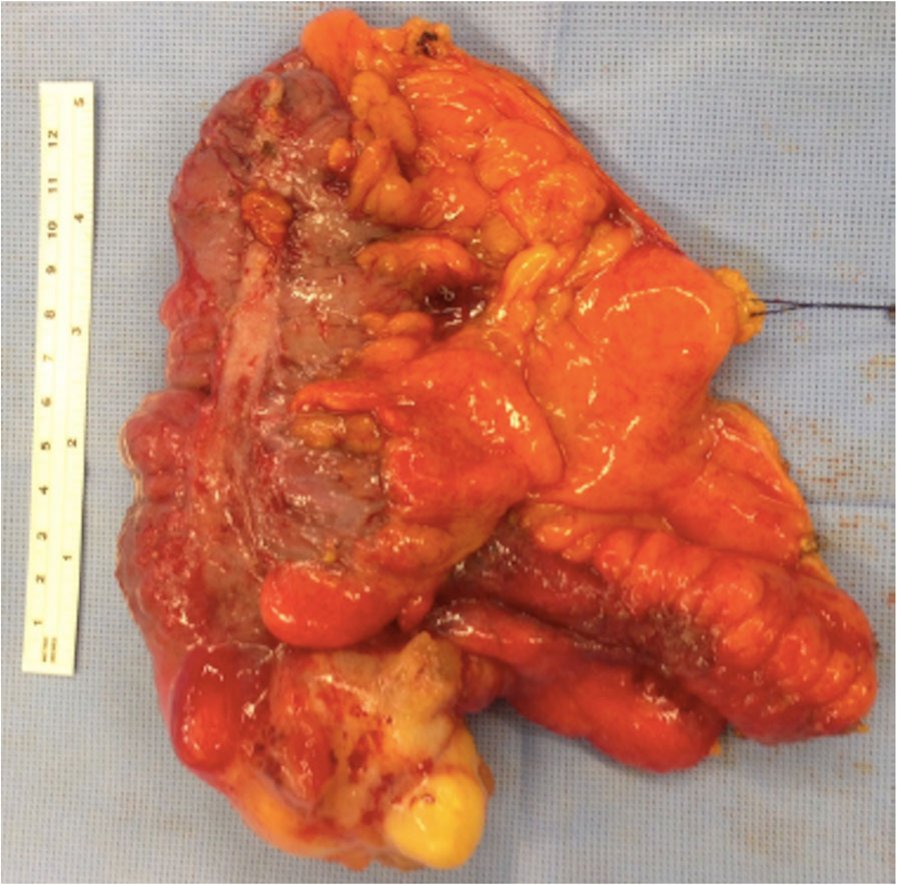
Surgical specimen from ileocolectomy to treat perforated appendiceal mucinous adenocarcinoma

In 2003, Misdraji and collaborators reclassified mucinous disease to low-grade mucinous neoplasia (LGMN) and mucinous adenocarcinoma (Figs. 5 and 6) [10].

### Clinical presentation and prognosis

**Fig. 7:**
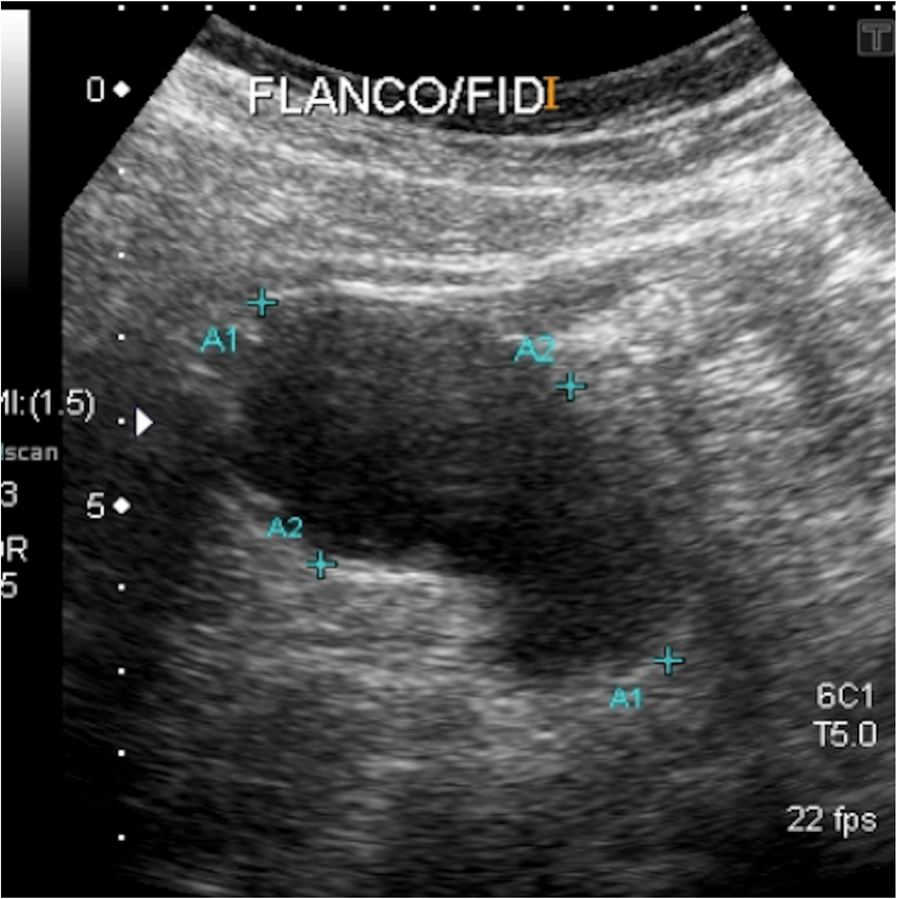
Dilated appendix, with a diameter of 3.5 cm compatible with appendiceal mucocele, in ultrasound

**Fig. 8:**
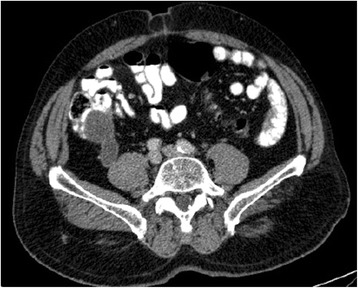
Axial computerized tomography view of a burst cecal appendix (up to 3.5 cm), with thin and regular walls, and no signs of densification of adjacent adipose tissue. This corresponds to the cystic formation already described in the ultrasound, compatible with mucocele of undetermined etiology. Appendectomy revealed well-differentiated mucinous adenocarcinoma, with invasion into of the muscularis mucosae

Performance and patient prognosis vary, and the diagnosis can be incidental, in a case of complicated acute appendicitis. However, preoperative diagnosis can be made by abdominal ultrasound with the finding of an appendix mucocele, indicating a dilated appendix filled with mucinous content (Figs. 7 and 8).

Eventually, in a similar clinical scenario, there is a condition in which the disease is located and restricted to the right lower quadrant of the abdomen. In those cases, mucin deposits can be found in the serosa, in the mesoappendix or extending to right iliac fossa. Those mucin lakes do not contain any neoplastic cells when submitted to pathological analysis [54].

**Fig. 9:**
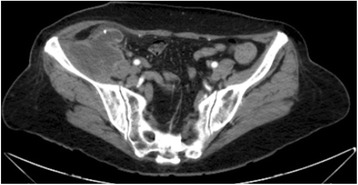
Axial computerized tomography view showing mucin lakes in a patient with low-grade appendiceal mucin adenocarcinoma limited to the right iliac fossa. Pathological analysis revealed extra-appendicular mucin without the presence of mucinous epithelium

**Fig. 10:**
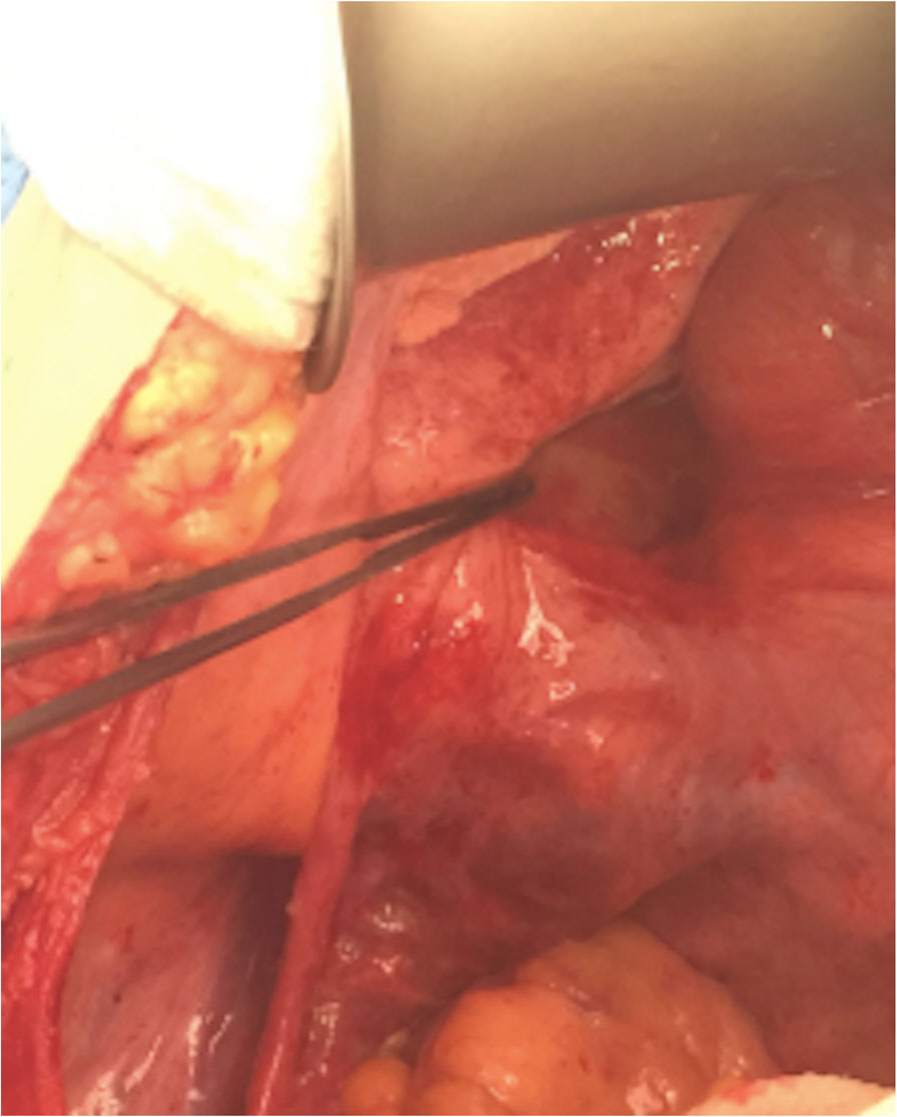
Intraoperative view of pericecal and retrocecal mucin. The disease was limited to the right iliac fossa. Pathological analysis analysis revealed no cells in the mucin

Yantiss and collaborators have studied the prognosis of patients with appendiceal mucinous neoplasms and mucin deposits in the right lower quadrant. Of 65 patients, 77% did not present cells in the mucin lakes while 23% had low-grade mucin epithelial cells. In follow-up after 52 months, 96% of the patients without cells in the mucin lakes were free the disease. In that group of patients showing no extra-appendicular mucinous epithelium, two of them developed peritoneal dissemination, but the appendix of those two individuals was not analyzed in the study [55]. Regarding patients presenting extra-appendicular mucinous epithelium, 33% evolved to pseudomyxoma peritonei. The study demonstrated excellent prognosis for patients with mucin dissemination in the right lower quadrant and no extra-appendicular mucinous epithelium [55] (Figs. 9 and 10).

Pseudomyxoma peritonei is considered by the UICC/AJCC and the WHO a malignant disease secondary to a carcinoma [56, 57].

The extension of a disease with extra-appendicular mucinous epithelium significantly affects patient prognosis. In a series of 184 patients, Carr and collaborators observed that mucinous disease restricted to the appendix has an indolent course. When the neoplasia is limited to the muscular mucosa, which is intact, it is called adenoma. When there is invasion of deeper layers or extra-appendicular extension, it is called carcinoma. Tumors that do not invade the muscular mucosa, but develop mucin deposits in the serosa without cells, are referred as uncertain potential for malignity [58].

Tumors characterized by neoplastic mucinous epithelium invading the muscular mucosa should be considered as high risk of peritoneum dissemination and classified and staged according to WHO and AJCC. Mucinous tumors with extra-appendicular dissemination of mucinous epithelium are considered by WHO low or high-grade mucinous adenocarcinoma, depending on the cytoarchitecture atypia.

Miner and collaborators analyzed histologic characteristics of appendiceal mucinous tumors presenting with peritoneal dissemination treated with surgical cytoreduction. In a retrospective series of 97 patients, complete cytoreduction was possible in 53% of the patients. Low-grade cytology was independently associated with longer disease-free survival. Some 90% of patients who lived an additional 10 years were diagnosed with low-grade tumors [59].

Traditional treatment for pseudo myxoma peritonei is composed of surgical cytoreduction associated with hyperthermic intraperitoneal chemotherapy (HIPEC) [60].

A retrospective analysis based on 2289 patients diagnosed with pseudo myxoma peritonei resulting from perforated appendiceal mucinous tumors showed the importance of associating complete macroscopic cytoreduction and HIPEC. The study reported 10 and 15 years survival in 63 and 59% of the cases respectively, with perioperative mortality of 2% [61].

### Appendiceal adenocarcinoma – Colonic-type

There are few studies on Colonic-type appendiceal adenocarcinoma. In a recent German multi-centric study about primary appendiceal carcinomas, 99 patients, with an average age of 64 years old, were identified. Those tumors had an average length of 3.27 cm, 72% were T3/T4 tumors, and 36.4% of them had lymph node metastasis. More than 60% were moderately differentiated, and more than 20% were poorly differentiated. Stage IV disease represented 23.2% of the cases, and overall survival was 47.5% for all stages. When compared to other more frequent primary appendiceal tumors (carcinoid and mucinous adenocarcinoma), the colonic-type has the higher incidence of lymph nodes metastasis [62, 63].

**Fig. 11:**
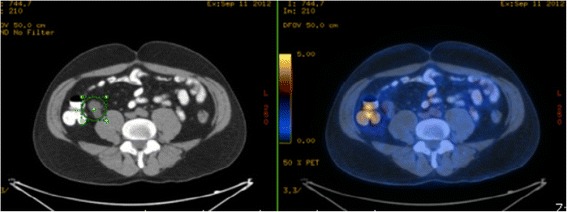
PET-CT of a patient subjected to appendectomy to treat acute appendicitis with incidental diagnosis of appendiceal tubular adenocarcinoma with signet-ring cell formation T3NXMX. In postoperative follow-up, CEA was elevated and the PEC-CT revealed pericecal lymphadenopathy

**Fig. 12:**
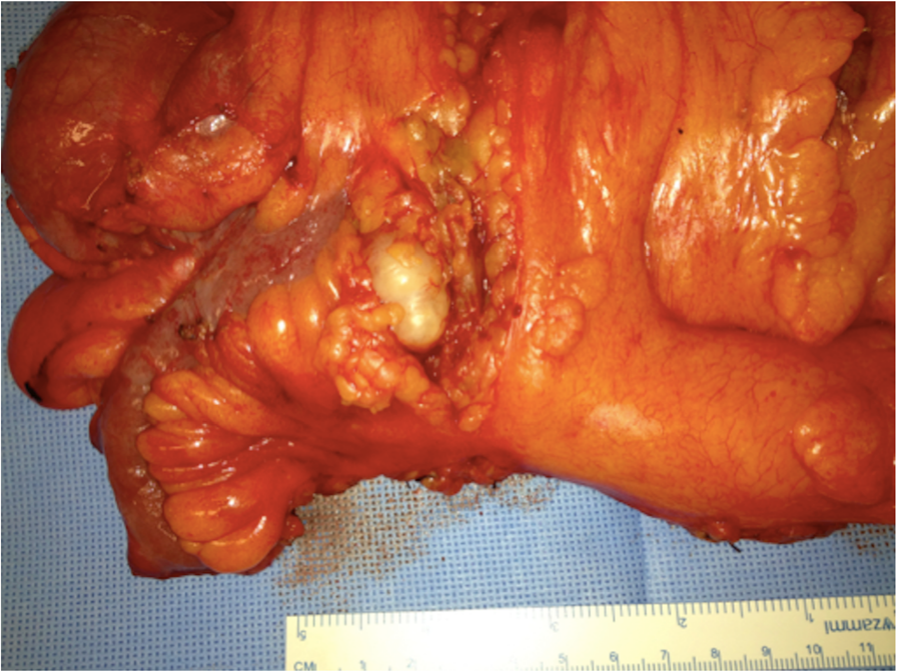
Product of ileocolectomy and/or right colectomy performed in same patient of Fig. 11, revealing pericecal lymphadenopathy. Margins in the caecum showed a 2.5 cm adenocarcinoma with signet-ring cell formation and metastasis in nine of the 14 dissected lymph nodes. pT3pN2

Such information reinforces the indication of any surgical intervention (appendectomy/ileocolectomy) required to treat and appendiceal inflammatory mass after conservative treatment (Figs. 11 and 12).

Literature is scarce when discussing patients diagnosed with adenocarcinomas with signet-ring cell formation or undifferentiated. The effects of therapeutic interventions (cytoreduction and systemic chemotherapy) in this group are unknown. In a series analyzing 142 patients diagnosed with undifferentiated tumors or with signet-ring cell formation (defined by the WHO as more than 50% of the cells in the tumor in a signet-ring formation), the average age was 52 years old, and 75% presented metastatic disease. In patients with metastatic disease, all of them had peritoneum involvement, and 36% also had it in the ovaries. The median survival rate was 2 years for patients diagnosed with undifferentiated adenocarcinomas and 2.5 years for those with adenocarcinomas with signet-ring cell formation. Patients who were submitted to surgical cytoreduction had a median recurrence-free survival of 1.2 years, and an overall survival of 4.2 years was achieved when patients underwent a complete cytoreduction. This surgical approach is also improves overall survival of patients with A oligometastatic peritoneal disease. Unfortunately, complete cytoreduction was only achieved in 21% of the patients [64].

## Conclusion

Hidden appendiceal neoplasm findings in post-operative appendectomy for acute appendicitis is a rare event, fortunately. However, its incidence in acute appendiceal inflammatory mass is not negligible. In our point of view all patients submitted upfront to a more conservative treatment (percutaneous drainage and antibiotics) should be submitted to interval appendectomy.
